# Still wanted—the mechanisms of consciousness!

**DOI:** 10.3389/fpsyg.2015.00005

**Published:** 2015-01-21

**Authors:** Jaan Aru, Talis Bachmann

**Affiliations:** ^1^Faculty of Mathematics and Computer Science/Faculty of Law, University of TartuTartu, Estonia; ^2^Faculty of Law, University of TartuTartu, Estonia

**Keywords:** consciousness, NCC, perception, state of consciousness, TMS, multivariate pattern analysis

Thirty years ago one of us proposed a theory of perception exemplified by the phenomenon of visual masking, based on neural mechanisms known to be responsible for contents and state of consciousness (Bachmann, 1984). Some of the colleagues labeled it “exotic,” perhaps trying to be polite by avoiding certain other adjectives. Be that as it may, within only a few years consciousness as a serious topic in mind sciences became firmly established (e.g., Baars, 1988; Crick and Koch, 1990). However, it can be argued that since then we have not learned much about the neural correlates of consciousness (NCC). Although many influential high-profile studies on consciousness have been published over the last 25 years, it is not clear how much of this research is directly relevant for understanding the neural basis of conscious experience (Aru et al., 2012a; de Graaf et al., 2012; Miller, 2015). The key reason for this pessimistic view is the following: many studies using various experimental paradigms have relied on the contrast between trials with and without conscious perception, but this contrast is not selective for revealing the NCC (Miller, 2007; Aru et al., 2012a; de Graaf et al., 2012). Rather, such contrast can always also lead to processes that in reality precede or follow conscious experience. As the majority of studies have been conducted with some variations of such contrastive analysis, it is hard to estimate how many of these studies are directly informative about NCC. Hence, although there are theories of consciousness that have made it to standard neuroscience textbooks, the puzzle of consciousness persists and the need for focused interdisciplinary attacks on the problem is as timely as ever.

One might think that we are overselling the problem, but contributions to the current research topic support our cautious standpoint. In particular, Pitts et al. (2014a) show that the P300, often declared to be the neural correlate of conscious access, is not observed when the subjects consciously perceive the target but it is task-irrelevant, i.e., when the subjects do not have to report the target (see also Pitts et al., 2014b). Also, Vidal et al. (2014) use an experimental paradigm of perceptual suppression through decreased contrast to demonstrate that local gamma band responses in several brain areas can increase while conscious perception is suppressed. Hence, increases in local gamma band responses do not reflect the NCC (see also Aru et al., 2012b). Finally, Ruhnau et al. (2014) review recent work illustrating that prerequisites of consciousness are not necessarily only local neural processes but rather depend on fluctuations of large-scale networks. Hence long-range integration across brain areas cannot be a specific marker of NCC. Although there will be definitely debates about these particular studies and results, the bottom line here is that the status of certain neural processes once thought to reflect the correlates of consciousness (such as the P300, local gamma band responses and long-range integration) has to be carefully reconsidered. (We kindly remind the readers that discussions about these studies can be carried out in the comments section of the respective articles.)

In addition to these studies showing that old results and theories have to be re-evaluated (Pitts et al., 2014a; Ruhnau et al., 2014; Vidal et al., 2014), the present research topic provided the following potential guidelines for consciousness research:

1. There are many different aspects of consciousness that should not be confounded with each other. In addition to the classic distinction between phenomenal, access and reflective consciousness (Block, 1995); Navajas et al. (2014) describe the need to distinguish between perceptual and contextual awareness. Among other things, future research should study possible differences in the qualitative experiences between these two varieties of consciousness.
2. Safavi et al. (2014) point out that for understanding a complex phenomenon like conscious experience it is necessary to consider data provided by various experimental techniques to assess brain activity on different spatial and temporal scales. The contributions to the present research topic adhere to this variety, as results from intracranial measurements (Vidal et al., 2014), fMRI (Wang and He, 2014), and EEG (Pitts et al., 2014a) are considered in the original research contributions to the research topic.
3. It is furthermore clear that one does not only want to measure consciousness and the associated processes, but also manipulate them. Tapia and Beck (2014) and de Graaf and Sack (2014) provide an update of how studies with TMS are contributing to the science of consciousness (see also Bachmann and Francis, 2013, for this purpose). Tapia and Beck (2014) describe the ways how combining and comparing traditional visual modal masking and TMS-masking can inform us about the putative mechanisms of visual consciousness, roles of feedforward and feedback processes, and also the possible meaning of alpha-frequency activity in mediating awareness. In addition to reviewing the pertinent TMS-masking research and commenting on other non-invasive brain stimulation methods for studying NCC, de Graaf and Sack (2014) describe another important use of TMS—creating artificially evoked phenomenology such as phosphenes. The contributions of this Research Topic are not alone in the recent works emphasizing the usefulness of stimulation methods for locating the NCC (Parvizi et al., 2012; Koubeissi et al., 2014).
4. From the data analysis perspective it is essential that the science of consciousness incorporates the advances in multivariate decoding of neural activity patterns (Haynes, 2009). Sandberg et al. (2014) review the basics of pattern analysis techniques and discuss their main contributions to studying the NCC.
5. We often seem to forget a very basic attribute of consciousness—its panmodality. We are conscious of sounds, lights, smells, etc. Thus, in order to get closer to NCC it is useful to investigate the neural correlates of intermodal and cross-modal processes of conscious experience. Wang and He (2014) extracted brain activity present during ongoing conscious flow of story experience regardless of input modality and describe the network underlying this modality-independent processing.
6. Furthermore, it is necessary that researchers studying the state of consciousness and contents of consciousness unite their forces to provide a complete account about how consciousness emerges from the neural processes (Hohwy, 2009; Bachmann and Hudetz, 2014). More specifically, it also may require cellular and sub-cellular level research and mechanistic level analysis in order to integrate state- and content-based approaches (Bachmann and Hudetz, 2014).
7. Finally, one has to stay optimistic, but at the same time also realistic. Miller (2014, 2015) argues that ultimately one does not want to find only the NCC but rather the neural constituents of consciousness. He makes it evident that we currently do not even have the strategy for approaching the neural constituents of consciousness. Let us then remark here that we currently have trouble even with the NCC.

It might be that for understanding the NCC we need more advanced tools for measuring and controlling neural processes. However, it is important to note that such sophisticated tools will not be sufficient for closing in on the mechanisms of consciousness due to the problem mentioned above: contrasting trials with and without conscious perception cannot reveal the NCC. Thus, given the accelerating pace of technical improvements it is necessary that the cognitive neuroscience community steps up the game too and asks: how can our experimental paradigms specifically target the neural basis of consciousness? The present research topic did not provide definite answers, but several promising directions of research have been envisaged and potential pitfalls pointed at.

## Conflict of interest statement

The authors declare that the research was conducted in the absence of any commercial or financial relationships that could be construed as a potential conflict of interest.
